# Patient-reported utilities in bilateral visual impairment from amblyopia and age-related macular degeneration

**DOI:** 10.1186/s12886-016-0234-0

**Published:** 2016-05-17

**Authors:** Elizabeth S. van de Graaf, Dominiek D. G. Despriet, Caroline C. W. Klaver, Huibert J. Simonsz

**Affiliations:** Department of Ophthalmology, Erasmus MC, University Medical Center Rotterdam, PO Box 2040, NL-3000 CA Rotterdam, The Netherlands; Department of Ophthalmology, Admiraal de Ruyter Hospital, PO Box 3200, NL-4380 DD Vlissingen, The Netherlands

**Keywords:** Patient-reported utilities, Bilateral visual impairment, Amblyopia, Age-related macular degeneration, Elderly

## Abstract

**Background:**

Utility of visual impairment caused by amblyopia is important for the cost-effectiveness of screening for amblyopia (lazy eye, prevalence 3–3.5 %). We previously measured decrease of utility in 35-year-old persons with unilateral persistent amblyopia. The current observational case–control study aimed to measure loss of utility in patients with amblyopia with recent decrease of vision in their better eye. As these patients are rare, the sample was supplemented by patients with bilateral age-related macular degeneration with similar decrease of vision.

**Methods:**

From our out-patient department, two groups of patients with recent deterioration to bilateral visual acuity less than Snellen 0.5 (bilateral visual impairment, BVI) were recruited, with either persistent amblyopia and age-related macular degeneration (AMB + AMD), or with bilateral age-related macular degeneration (BAMD). To measure utility, the time trade-off method and the standard gamble method were applied through interviews. Correlations were sought between utility values and visual acuity, age and Visual Function Questionnaire-25 scores.

**Results:**

Seventeen AMB + AMD patients (mean age 72.9 years), and 63 BAMD patients (mean age 79.6 years) were included in the study. Among AMB + AMD, 80 % were willing to trade lifetime in exchange for cure. The overall mean time trade-off utility was 0.925. Among BAMD, 75 % were willing to trade, utility was 0.917. Among AMB + AMD, 38 % accepted risk of death in exchange for cure, overall mean standard gamble utility was 0.999. Among BAMD, 49 % accepted risk of death, utility was 0.998. Utility was not related to visual acuity but it was to age (*p* = 0.02).

**Conclusion:**

Elderly patients with BVI, caused by persistent amblyopia and age-related macular degeneration (AMD) or by bilateral AMD, had an approximately 8 % loss of TTO utility. Notably, the 8 % loss in elderly with BVI differs little from the 3.7 % loss we found previously in 35-year-old persons with unilateral amblyopia with good vision in the other eye. The moderate impact of BVI in senescence could be explained by adaptation, comorbidity, avoidance of risk and a changed percept of cure.

## Background

Amblyopia (lazy eye) is not congenital, but results from strabismus (squint) or from anisometropia (unequal refractive power of the eyes), or, rarely, from deprivation of the eye, caused by congenital cataract, for instance. It has a prevalence of 3–3.5 %, depending on population and criteria [1]. In developed countries, most children with amblyopia are detected by pre-school screening by measurement of the visual acuity at age four or five. Amblyopia can be treated before the age of seven. If insufficiently treated or unresponsive to treatment, the eye with persistent amblyopia retains a Snellen visual acuity (VA) of less than 0.5 and patients will be unable to read with that eye during the rest of their life.

The cost-effectiveness of screening programmes for amblyopia has been the subject of several reports [2, 3]. In a report by Carlton et al. [2] the cost-effectiveness of vision screening up to the age of 4–5 years was examined. They concluded that the cost-effectiveness of screening for amblyopia depends primarily on the long-term utility effects of unilateral vision loss caused by persistent amblyopia and that there is currently no evidence of loss in utility that would render any screening to be cost-effective.

In a previous study, we examined the decrease in quality of life in a historic cohort of adults with amblyopia by a questionnaire [4]. We subsequently measured the loss of utility due to unilateral persistent amblyopia in the same historic cohort of adult persons with unilateral persistent amblyopia, 35–40 years old, who had, 30–35 years previously, been treated for amblyopia in Waterland, a rural region north of Amsterdam [5]. This historic cohort formed an almost random sample, as almost all children with amblyopia and/or strabismus were referred and treated by the only ophthalmologist and only orthoptist in Waterland at the time. Almost half of them could be contacted 30–35 years later. Seventy percent of these persons (*N* = 135) were willing to trade lifetime in exchange for cure, i.e. perfect vision, measured by the Time Trade-Off (TTO) method. They had, on average, a utility of 0.963, in other words, a loss of utility of 3.7 %. The loss of utility correlated with the current visual acuity of the amblyopic eye [5].

Occasionally, persons with unilateral persistent amblyopia also lose the function of their better eye, usually at an older age. Among 6-years-olds with persistent amblyopia, the period of time at the end of their life with bilateral visual impairment (BVI) defined in that in the current study as Snellen VA of < 0.5 in both eyes is, on average, fifteen-and-a-half months against eight months in healthy six-years-olds, according to our longitudinal study performed in a population cohort of elderly [6].

In the current study we measured the utility in the patients with unilateral persistent amblyopia (either detected too late, insufficiently treated by glasses or patching, or unresponsive to treatment) who recently suffered a loss of vision in the better eye due to age-related macular degeneration (AMB + AMD). As these patients are rare, the sample was supplemented by patients who had bilateral age-related macular degeneration (BAMD) and also recently suffered a loss of vision in the better eye.

## Methods

Patients from the ophthalmology out-patient clinic, Erasmus MC, Rotterdam were recruited from Autumn 2009 till Spring 2011 to form two groups of consecutive patients. Included in the study were those with amblyopia with a VA of less than 0.5 (Snellen) with a recent reduction of vision in the better eye due to AMD, the AMB + AMD group, and those with existing AMD in one eye and recent reduction of vision in the better eye due to AMD (both eyes Snellen VA < 0.5), the BAMD group. Data were collected by patients-interviews for approximately thirty minutes by the first author. Patients who could not be interviewed because of illness or inability to speak Dutch were excluded. The decrease in VA had occurred within six months before the date of interviewing the patients. As the patient’s eyes were treated with injections of bevacuzimab for AMD, sometimes the VA of the deteriorated eye had improved before the interview. Diagnostic VA measurements had been performed by optometrists, ophthalmologists and residents, and were taken from the patient’s medical record for this study.

Utility is the preference or value (ranging from 1.0, perfect health, to 0.0, death) the patient assigns to his present health state including the disability he/she experiences, like bilateral visual impairment in this study. Direct patient’s preferences about the health state of BVI were obtained by the methods of Time Trade-Off (TTO) and Standard Gamble (SG). Both methods were based on the same search procedure and forced search alternatives as previously used [5].

TTO utilities were elicited by posing patients the question: “Suppose there is a hypothetical medicine that restores perfect vision in both eyes, but it would shorten your remaining lifetime; would you give up … (lifetime-alternative) in exchange for perfect vision”. Forced TTO search alternatives were “no time”, “1 day”, “1 week”, “1 month”, “3 months”, “6 months”, “1 year”, “2 years” and “5 years”. SG utilities were elicited by posing patients the question: “Suppose there is a hypothetical surgery that restores perfect vision in both eyes, but here is an immediate risk of death; would you accept such risk of …(risk-alternative) in exchange for perfect vision”. Forced SG search alternatives were “no risk”, “risk of 1:20,000”, “1:10,000”, “1:1,000”, “1:200”, “1:100” and “1:50”.

The patients were in addition asked to choose, out of three, the most troubling aspect of the affected visual eye function: Blurred vision in the worse eye, blurred vision in the center of the visual field of the better eye or lack of depth vision. The measured utility values were compared with the VA in the better eye to ascertain whether the utility values were associated to VA.

Finally, all patients filled out the National Eye Institute Visual Function Questionnaire-25 with supplement (VFQ-25) which is a general vision quality-of-life instrument [7].

Statistics were performed in Excel 2003 and SAS 9.2, the significance test of the group-difference and the correlation between the VFQ-25 scores and utility value in SPSS (version 13).

## Results

### Study population

Patients were recruited from Autumn 2009 till Spring 2011 from the ophthalmology out-patient clinic, at the Erasmus Medical Center. The 17 AMB + AMD patients were 35 % male and had a mean age of 72.9 ± 10.4 and median age of 76 at the time of the interview. The 63 BAMD patients were 38 % male and had a mean age of 79.6 ± 7.2 and median age of 81 at the time of the interview. The age difference, 72.9 for the AMB + AMD against 79.6 for BAMD, was significant: P = 0.031 (two-tailed) on the non-parametric Mann–Whitney test. At the beginning of the interview the patients were asked how long they expected to live. The self-estimated life expectancy for the AMB + AMD-group was 82.1 and for the BAMD-group 89.3.

The 80 patients were interviewed, in 13 cases face-by-face immediately after or before their ophthalmologic treatment at the Erasmus Medical Center or during a visit at their home and in 67 by phone, within a week after their ophthalmologic consult at the Erasmus Medical Center, taking approximately 30 min. In the AMB + AMD group, one patient was excluded because of illness. No patient refused the interview. In the BAMD group, three patients were excluded, two because of illness and one because of the inability to speak Dutch.

**Fig. 1:**
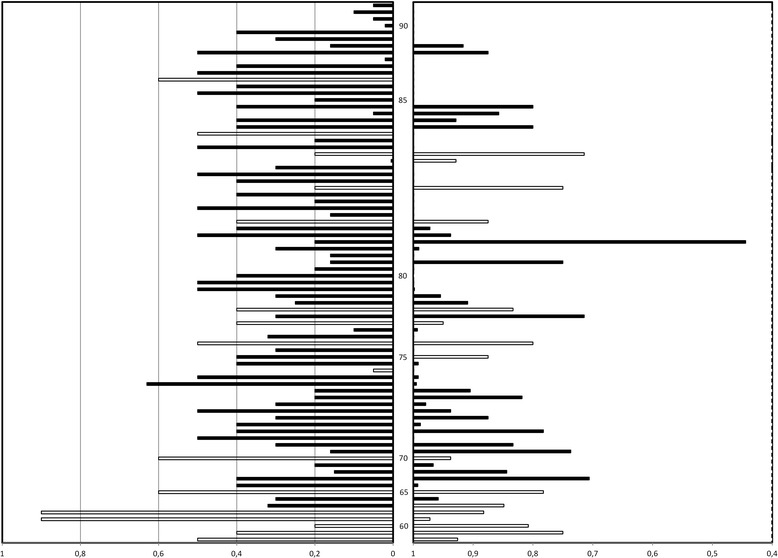
Distribution of the VA of the recently deteriorated eye (left bars) and TTO utility values (right bars) from all patients, listed by age. The 80 patients are listed from age 56 (bottom) to age 96 (top) on the ordinate. The centre column represents the age of the youngest patient of each five-year age group. In other words, the youngest patients in the sixty to sixty-four year age group is marked sixty. The open bars represent the AMB + AMD patients, the black bars represent the BAMD patients. Left bars represent Snellen VA, from worst (right) to best (left). Two patients with unilateral persistent amblyopia were later found to have VA of 0.9 in the amblyopic eye. Distribution of TTO utility from all patients is given at the right. The value at the right side of the bar represents the TTO utility, so the length of the bar depicts the difference between 1 and the TTO utility, which indicates the loss of TTO utility. No bar means that the patient was not willing to trade any remaining lifetime for cure

The distribution of the VA of the recently impaired eye at the time of the interview from all 80 patients is shown in Fig. 1 (left bars). The patients are listed by age, from 56 to 96 years, at the time of the interview.

### Utility values

TTO utility value was equal to: 1 - traded lifetime / self-estimated life expectancy. Mean TTO utility for AMB + AMDs was 0.925, and for BAMDs 0.917 (Table 1).

**Table 1:** TTO and SG results from the patient groups

	Time trade-off					
	Traded lifetime			Utility value		
Groups	N	%	Mean	Mean	Sd	Log. Mean
AMB + AMD	12	80	16 months	0.925	0.09	0.946
BAMD	41	75	14 months	0.917	0.11	0.943
	Standard gamble					
	Accepted death risk			Utility value		
Groups	N	%	Mean	Mean	Sd	Log. Mean
AMB + AMD	6	38	0-1:10,000	0.999	0.0004	0.999
BAMD	27	49	0-1:50,000	0.998	0.004	0.999

Note: Mean traded lifetime, mean accepted death risk, mean Time Trade-Off utility value and mean Standard Gamble utility value are averaged over all patients per group. “AMB + AMD” denotes amblyopia and age-related macular degeneration, “BAMD” bilateral age-related macular degeneration, “Sd” standard deviation, “Log. Mean” logarithmic mean

Mean traded lifetime and TTO mean utility values were approximately the same for both groups (averaged over all patients per group) (Table 1). Logarithmic mean TTO utility was higher for both groups (averaged only over patients per group who were willing to trade lifetime) (Table 1). The standard deviation given does not reflect true variation, as the distribution is skewed and the utility value cannot exceed 1.0. Significant correlation was found for all patients (*N* = 80) between TTO utility and age (linear regression with bootstrap method, p < 0.001). Most of the oldest persons were unwilling to give up any lifetime. The TTO utility values from all 80 patients are also represented in Fig. 1 (right bars). Figure 1 shows that older patients were less willing to give up lifetime (top right). There was no significant relation between TTO utility and the patients’ VA (left bars), but there was between TTO utility and age for the BAMD-group.

SG utility value was equal to: (1 - accepted risk of death). Of the AMB + AMDs, only 38 % accepted risk of death and of the BAMDs, 49 % accepted such risk. The mean risk of death they accepted was 1 in 10,000 and 1 in 50,000, respectively (Table 1). The risk of death alternatives that were accepted ranged from 1 in 50 (two patients: aged over 80 years, each with BAMD) to 1 in 20,000. Mean SG utility for the AMB + AMD-group was 0.999, and for the BAMD-group 0.998. Logarithmic mean SG utility, averaged over the patients per group only who accepted risk of death, was 0.999 for both groups.

**Fig. 2:**
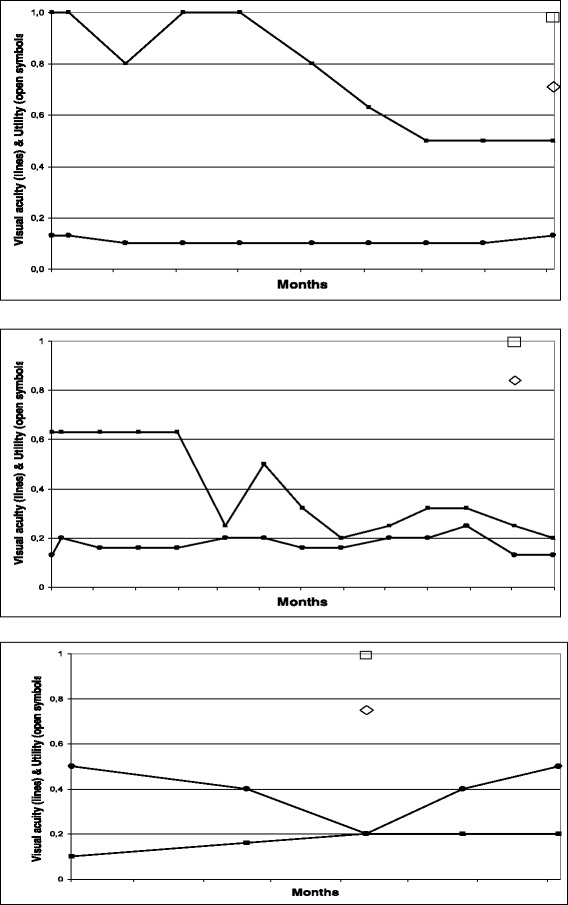
TTO utility value (diamond), SG utility value (square) and consecutive visual acuity of both eyes set against time in months as illustrated by three patients. The abscissa represents time VA measured in months. Scale of TTO utility, SG utility and visual acuity are represented by the ordinate. It runs from 0.0 to 1.0, signifying either Snellen VA, measured TTO utility or measured SG utility. Open diamonds and open squares represent TTO and SG utility at the time of the interview. VA of the right eye is represented by filled-in circles, VA of the left eye by filled-in squares, connected by lines. VA was measured in the months preceding the interview and/or after the interview. The association between visual acuity and the decrease in visual acuity on the one hand, and measured utility on the other hand, is very weak

The inclusion criterion of a decrease in Snellen VA to less than 0.5 could not be upheld in one patient who had Snellen VA of 0.5. Another patient had a known amblyopia with Snellen VA 0.8 when he entered into the study. Later, after full correction with glasses, they were both found to have improved to Snellen VA of 0.9 in the amblyopic eye. Some patients had had an abrupt decrease of VA (Fig. 2a) whereas others had a slow decrease. It was difficult to ascertain the exact date of the decrease of VA: There was a delay between the occurrence of acuity decrease and the diagnosis by the ophthalmologist, in addition to the waiting time for the appointment with the general practitioner and for that with the ophthalmologist (Fig. 2b). Finally, as the patient’s eyes were injected with bevacuzimab for AMD progression, the VA of the deteriorated eye had improved in six patients around or after the time of the interview (Fig. 2c).

All patients chose, out of three possibilities, the visual function that disturbed them most. Patients of both groups found blurred vision in the center of the visual field of the better eye the most troubling disturbance: 77 % of the AMB + AMD and 52 % of the BAMD patients. Blurred vision in the worse eye was the second most troubling visual function for the AMB + AMDs (17 %) and the BAMDs (42 %). Metamorphopsia, i.e. a wrinkled image caused by AMD was mentioned by some patients during the interview.

### Comparison with VFQ-25

The questions of the VFQ-25 associated with patients’ low quality of life concerned reading of normal print of books and newspapers, small print on groceries and subtitles of television programs, driving a car, writing, and hobbies like embroidery and stitching work. The lifetime which the patients were willing to trade had only a modest correlation with the VFQ-25 A2 scale on vision health: *P* = .077 with Spearman correlation -.240. It was not correlated with the VFQ-25 A1 scale on general health, although co-morbidities like diabetes or heart failure were mentioned during the patient-interviews. Visual aids were used in order to read: most patients used hand-held magnifiers, some used plate magnifiers for newspapers and some used telescope glasses for subtitles.

## Discussion

Elderly patients with BVI, either caused by persistent amblyopia and AMD, or caused by bilateral AMD had an overall mean TTO utility value of approximately 0.92, in other words approximately 8 % loss of TTO utility. Previous studies of utility of BVI found considerably lower TTO values, of 0.73 or less [8, 9], but these authors applied either indirect methods (indexed health states evaluated by predetermined search alternatives) or clinicians’ and community members’ derived preferences. In view of the moderate loss of utility we found, together with the rare occurrence of BVI in patients with amblyopia, it seems that the impact of loss of function of the better eye on the cost-effectiveness of screening for amblyopia is minor.  The 8 % utility loss in elderly with BVI we found in this study differs little from the utility loss of 3.7 % we found previously in young adult persons with unilateral persistent amblyopia with good vision in the other eye [5]. That condition occurs much more frequently, approximately 0.9 % of the population in the RAMSES birth cohort study [1] and therefore its impact on the cost-effectiveness of screening for amblyopia is large.

A similar fraction of the AMB + AMD and the BAMD patients, viz. 80 and 75 %, respectively, was willing to trade lifetime: on average sixteen months vs. fourteen months. The average age was 72.9 years in the AMB + AMD group and 79.6 years in the BAMD group. The difference in age between the two groups (significance p = 0.031; Mann–Whitney test) was to be expected as BVI occurs earlier in persons with unilateral persistent amblyopia than in healthy persons [6]. Both groups estimated their remaining lifespan at approx. 10 years. In the general Dutch population, a person of 72.9 years lives 86 years in total, and a person aged 79.6 years lives 88.5 years, on average.

With the SG method, only 38 % of the AMB + AMD and only 49 % of the BAMD patients accepted risk of death in exchange for cure. Both groups of elderly patients with BVI had an overall (including all who accepted no risk of death) mean SG utility value of approximately 0.999, in other words approximately a 0.1 % loss of SG utility. Only two patients, aged over 80 years with BAMD, accepted a risk of death of 1 in 50. In our previous study, in 35-year-old persons with unilateral amblyopia, 37 % accepted risk of death [5]. Considering the fact that the SG method implies risk of immediate death, it is understandable that most patients will not accept such a risk [10]. Similar to Brown et al. [11], we found that the TTO utility value was lower than that from SG for the disability of BVI. The disturbance that most affected the patients’ visual function was blurred vision in the center of the visual field of the recently deteriorated eye which was in accordance with the evaluation of their vision-related quality of life by the VFQ-25. The day-to-day tasks that the patients found difficult to perform were reading of normal and small print, driving a car and writing; these activities depend most on good central vision.

 The study has two limitations. The first one was the inability to derive utilities from patients in their pre-BVI health state with the consequence that we could not compare patients’ pre-BVI and BVI utility values. Secondly, the inclusion criterion of a decrease to Snellen VA <0.5 could not be upheld in rare patients who were found to have a major improvement in Snellen VA in the amblyopic eye. Six other patients had slightly higher acuity than threshold, namely Snellen VA of 0.6 in the better eye.

As said, in an earlier study we found that patients with unilateral visual impairment from persistent amblyopia, approximately 35 years old, had 3.7 % loss of TTO utility [8]. This may seem a small difference as compared to the 8 % loss in the 75-years-old patients with BVI in the current study.

We think the moderate loss of utility caused by BVI in 75-years-old patients, compared with that caused by unilateral persistent amblyopia –with good vision in the other eye- in 35-years-old patients can be explained by adaptation, avoidance of risk, comorbidity and a changed percept of cure. Also, response shift does occur when eliciting utilities from old patients.

First, adaptation is an important coping mechanism of patients to diminish the impact of chronic impairments, described in clinical rehabilitation studies [12]. Adaptation is age-dependent: elderly patients have had longer time than adult patients of younger age to adapt to an identical chronic disability, like visual function loss. Hence, the 75-years-old AMB + AMD patients have adapted more than the 35-years-old amblyopes to visual deterioration and its resulting functional restrictions. In addition, hedonic adaptation could have occurred: The unilateral amblyopia and unilateral AMD patients were, in first instance, shocked by the deterioration in the better eye due to the AMD but after a while would have accepted that and its accompanying restrictions.

Secondly, patients older than 85 year did not want to trade lifetime (see Fig. 1) or to accept risk of death, compatible with the phenomenon of risk avoidance at old age [13]. Consistent with this observation is the correlation between age and TTO utility in all patients (*p* < 0.001) that we found.

Thirdly, outcomes suggested a relation between TTO value, VFQ-25A1 general health scale and VFQ-25A2 vision health. Patients who had low vision in both eyes but still had a good general health, i.e. without comorbidity, seemed more willing to trade lifetime. This could, however, not be confirmed statistically. Other studies have also indicated that patients with worse general health due to additional diseases like diabetes or heart failure, are less willing to trade lifetime [14, 15].

Fourthly, healthy persons of approximately 37 years with unilateral amblyopia have a higher demand of their normal functioning within their career than elderly patients who are mostly retired. The younger adults have to be able to drive a car and do their jobs or do the household; they therefore have a higher percept of cure (“internal standard of cure”) than the elderly patients with BVI. 

Finally, some patients voiced a decreased relevance of quality of life in general at old age, analogous to a response shift, i.e. a re-conceptualization of quality of life in general. Patients seemed to accept the forthcoming deterioration of their health, including their vision, when they were unwilling to give up lifetime [16]. The similar phenomenon occurs also in the acceptance of pain among elderly [17].

## Conclusions

Elderly patients with BVI, caused by unilateral persistent amblyopia and AMD or by bilateral AMD, had an approximately 8 % loss of TTO utility. This loss in elderly with BVI differs little from the 3.7 % loss in 35-year-old persons with unilateral amblyopia –with good vision in the other eye- we found in a previous study, indicating a lessened impact of visual impairment in senescence.

## Ethics approval and consent to participate

A statement of no objection was obtained from the Medical Ethical Committee of the Erasmus MC to perform the study. Patients gave their informed oral consent before participating in the study.

## Consent for publication

All authors gave consent to publish the manuscript.

## Availability of data and materials

All data will be made available upon request.

## Abbreviations

AMB + AMD: amblyopia and age related macular degeneration
AMD: age-related macular degeneration
BAMD: bilateral age-related macular degeneration
BVI: bilateral visual impairment
SG: standard gamble
TTO: time trade-off
VA: visual acuity
